# Can Combined Screening of Ultrasound and Elastography Improve Breast Cancer Identification Compared with MRI in Women with Dense Breasts-a Multicenter Prospective Study

**DOI:** 10.7150/jca.43326

**Published:** 2020-04-06

**Authors:** Lu-Ying Gao, Yang Gu, Wen Xu, Jia-Wei Tian, Li-Xue Yin, Hai-Tao Ran, Wei-Dong Ren, Yu-Ming Mu, Jie-Ying Zhang, Cai Chang, Jian-Jun Yuan, Chun-Song Kang, You-Bin Deng, Hui Wang, Xiao-Yan Xie, Bao-Ming Luo, Sheng-Lan Guo, Qi Zhou, En-Sheng Xue, Wei-Wei Zhan, Tong Jiao, Qing Zhou, Jie Li, Ping Zhou, Pin-Tong Huang, Hong-Yuan Xue, Chun-Quan Zhang, Man Chen, Xiang-Xiang Jing, Ying Gu, Jian-Feng Guo, Hong-Yu Ding, Jin-Feng Xu, Wu Chen, Li Liu, Yu-Hong Zhang, Hong-Qiao Wang, Zhong-Ping Mu, Jian-Chu Li, Hong-Yan Wang, Yu-Xin Jiang

**Affiliations:** 1 Department of Ultrasound, Peking Union Medical College Hospital, Chinese Academy of Medical Sciences and Peking Union Medical College, Beijing 100730, China; 2 Department of Ultrasound, the Second Affiliated Hospital of Harbin Medical University, Harbin 150086, China; 3 Cardiovascular Ultrasound and Non-invasive Cardiology Department, Key Laboratory of Ultrasound in Cardiac Electrophysiology and Bio-mechanics of Sichuan Province, Sichuan Academy of Medical Sciences & Sichuan Provincial People′s Hospital, Chengdu 610072, China; 4 Department of Ultrasound, the Second Affiliated Hospital of Chongqing Medical University, Chongqing 400010, China; Chongqing Key Laboratory of Ultrasound Molecular Imaging, Chongqing 400010, China; 5 Department of Ultrasound, Shengjing Hospital of China Medical University, Shenyang 110004, China; 6 Department of Ultrasonography, the First Affiliated Hospital of Xinjiang Medical University, Urumqi 830054, China; 7 Department of Radiology, Cancer Institute &Hospital, Chinese Academy of Medical Sciences, Beijing 100730, China; 8 Department of Medical Ultrasound, Fudan University Shanghai Cancer Center & Department of Oncology, Shanghai Medical College, Fudan University, Shanghai 200032, China; 9 Department of Ultrasonography, Henan Provincial People′s Hospital, Zhengzhou 450003, China; 10 Department of Ultrasound, Shanxi Academy of Medical Science, Dayi Hospital of Shanxi Medical University, Taiyuan 030032, China; 11 Department of Medical Ultrasound, Tongji Hospital, Tongji Medical College of Huazhong University of Science and Technology, Wuhan 430030, China; 12 Department of Ultrasound, China-Japan Union Hospital of Jilin University, Changchun 130033, China; 13 Department of Medical Ultrasonics, Division of Interventional Ultrasound, Institute of Diagnostic and Interventional Ultrasound, the First Affiliated Hospital of Sun Yat-sen University, Guangzhou 510080, China; 14 Department of Ultrasound, the Sun Yat-sen Memorial Hospital, Sun Yat-sen University, Guangzhou 510120, China; 15 Department of Ultrasonography, First Affiliated Hospital of Guangxi Medical University, Nanning 530021, China; 16 Department of Medical Ultrasound, the Second Affiliated Hospital, School of Medicine, Xi'an Jiaotong University, Xi'an 710004, China; 17 Department of Ultrasound, Union Hospital of Fujian Medical University, Fujian Institute of Ultrasound Medicine, Fuzhou 350001, China; 18 Department of Ultrasound, Ruijin Hospital, Shanghai Jiaotong University, School of Medicine, Shanghai 200025, China; 19 Department of Ultrasound, Tianjin Union Medicine Hospital, Tianjin 300121, China; 20 Department of Ultrasonography, Renmin Hospital of Wuhan University, Wuhan 430060, China; 21 Department of Ultrasound, Qilu Hospital, Shandong University, Jinan 250012, China; 22 Department of Ultrasound, the Third Xiangya Hospital of Central South University, Changsha 410013, China; 23 Department of Ultrasound, the Second Affiliated Hospital of Zhejiang University, Hangzhou 310000, China; 24 Department of Ultrasound, Hebei General Hospital, Shijiazhuang 050000, China; 25 Department of Ultrasound, the Second Affiliated Hospital of Nanchang University, Nanchang 330006, China; 26 Department of Ultrasound Medicine, Tongren Hospital, Shanghai Jiao Tong University School of Medicine, Shanghai 200336, China; 27 Department of Ultrasound, Hainan Provincial People's Hospital, Haikou 570311, China; 28 Department of Ultrasonography, the Affiliated Hospital of Guizhou Medical University, Guiyang 550004, China; 29 Department of Ultrasound, the Affiliated Suzhou Hospital of Nanjing Medical University, Suzhou Municipal Hospital, Suzhou 215001, China; 30 Department of Ultrasonography, Qianfoshan Hospital of Shandong University, Jinan 250014, China; 31 Department of Ultrasound, Shenzhen People's Hospital, the Second Clinical Medical College of Jinan University, Shenzhen 518020, China; 32 Department of Ultrasound, the First Hospital of Shanxi Medical University, Taiyuan 030001, China; 33 Department of Ultrasound, Peking University Shenzhen Hospital, Shenzhen 518036, China; 34 Department of Ultrasound, the Second Hospital of Dalian Medical University, Dalian 116027, China; 35 Department of Ultrasound, the Affiliated Hospital of Qingdao University, Qingdao 266003, China; 36 Department of Ultrasound, Maternal and Child Health Care Hospital Affiliated to Medical University of Anhui, Anhui Province Maternal and Child Health Hospital, Hefei 230001, China

**Keywords:** Breast cancer, Dense breast, Breast Imaging Reporting and Data System (BI-RADS), Elastography, Magnetic resonance imaging (MRI), Ultrasound (US)

## Abstract

**Objectives**: To assess the performance of elastography (ES) and ultrasound (US) in predicting the malignancy of breast lesions and to compare their combined diagnostic value with that of magnetic resonance imaging (MRI).

**Materials and Methods**: The study prospectively enrolled 242 female patients with dense breasts treated in 35 heath care facilities in China between November 2018 and October 2019. Based on conventional US and elastography, radiologists classified the degree of suspicion of breast lesions according to the US Breast Imaging Reporting and Data System (BI-RADS) criteria. The diagnostic value was compared between US BI-RADS and MRI BI-RADS, with pathological results used as the reference standard.

**Results**: The results demonstrated that irregular tumor shape, a nonparallel growth orientation, indistinct margins, angular contours, microcalcifications, color Doppler flow and ES score on US imaging were significantly related to breast cancer in dense breasts (P=0.001; P=0.001; P=0.008; P<0.001; P=0.019; P=0.008; P=0.002, respectively). The sensitivity, specificity, PPV, NPV, accuracy and AUC of US BI-RADS category were 94.7%, 90.7%, 95.8%, 88.0%, 93.4% and 0.93 (95%CI, 0.88-0.97), respectively, while those of MRI BI-RADS category were 98.2%, 57.5%, 84.3%, 83.3%, 86.0% and 0.78 (95%CI, 0.71-0.85), respectively. MRI BI-RADS showed a significantly higher sensitivity than US BI-RADS (98.2% vs 94.7%, P=0.043), whereas US BI-RADS showed significantly higher specificity (90.7% vs 57.5%, P<0.001). US BI-RADS showed better diagnostic efficiency in differentiating nodules in dense breasts than MRI BI-RADS (AUC 0.93 vs 0.78, P<0.001).

**Conclusion**: By combining the use of ES and conventional US, US BI-RADS had better diagnostic efficiency in differentiating nodules in dense breasts than MRI. For the diagnosis of malignant tumors in patients with dense breasts, MRI and US BI-RADS can be used as supplemental diagnostic tools to detect lesions, with US BI-RADS considered the preferred adjunctive resource.

## Introduction

Mammography is a standard screening test that has been proven to reduce breast cancer-related mortality 1,2. However, dense glandular structures reduce the sensitivity of mammography, causing delayed diagnosis and worse outcomes 3. Ultrasound and magnetic resonance imaging (MRI) as adjuncts to mammography can aid in assessing breast lesions in dense breasts. US is a commonly used modality for detecting early breast cancers in dense breasts 4,5. However, the sonographic appearance of benign and malignant nodules overlaps to some extent, causing overtreatment biopsies that are a major limitation of US. US elastography (ES) is a new technique that improves the diagnostic value of US 6,7. ES has been incorporated into the fifth edition of the Breast Imaging Report and Data System (BI-RADS) of the American College of Radiology (ACR), which suggests the risk stratification of breast lesions based on suspicious conventional US features and elastography 8.

MRI has been considered the most sensitive screening modality for breast cancer. However, its average specificity is relatively low and varies according to the tumor indications 9. A previous study performed in Korea showed that the addition of elastography and color Doppler US to B-mode US can increase the PPV of screening US in women with dense breasts 10. However, they did not include MRI information and failed to compare the diagnostic value of US and MRI for lesions in dense breasts. The objective of this study was to explore the potential of combining US and US elastography and compare the diagnostic value of US and MRI for predicting breast cancer in dense breasts.

## Materials and Methods

### Patients

**Figure 1:**
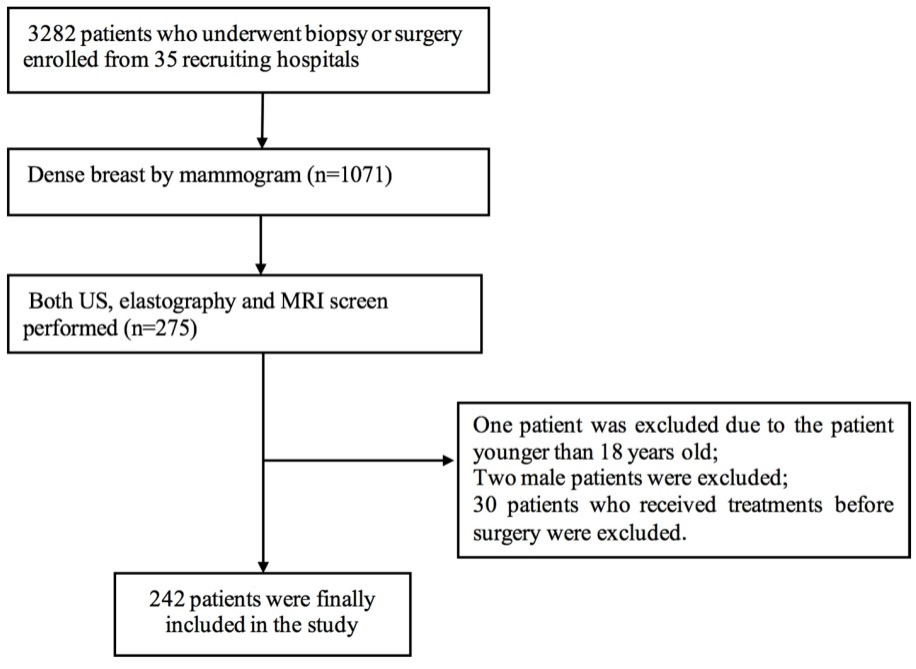
Flow chart of patient selection

This was a multicenter study conducted at regional medical centers in China, including 35 hospitals from 23 different provinces. All hospitals completed real-name registration on the website (www.nuqcc.cn) and a data survey after approval. The study prospectively enrolled 3292 patients with breast lesions who underwent biopsy or surgery at the 35 hospitals between November 2018 and October 2019. The following inclusion criteria were applied: (1) female patients older than 18 years of age; (2) patients assessed as having dense breast by mammography; and (3) patients for whom conventional US, elastography and MRI screening were performed. Patients who received treatment before surgery were excluded. A total of 242 patients were finally included [Figure 1]. All of these patients underwent breast ultrasound examination prior to core needle biopsy or surgical pathology. The final pathologic results were considered the diagnostic gold standard. The clinical features of the patients were recorded.

### Breast examination and prospective evaluation

All US examinations were performed with Resona7 or 8 devices (Mindray Medical, Shenzhen, China) equipped with 5-14 MHz linear-array transducers. US images were prospectively evaluated by 35 radiologists who were experienced in breast US and were blinded to the patient clinical data. First, conventional US images of the lesions were obtained, including B-mode US and color Doppler images. The tumor size, shape, echogenicity, growth orientation, margin, and contour, the presence of architectural distortion, the presence of duct ectasia, acoustic shadowing and microcalcifications were evaluated by B-mode US. Vascularity was classified into 4 patterns (no flow, minimal, moderate, or marked) by color Doppler flow 11. After conventional US, elastography images were generated by the same radiologists. Each lesion was assigned an elasticity score according to a 5-point scoring system 12. Based on B-mode US, color Doppler and elastography, the radiologists classified the degree of suspicion of breast lesions according to the BI-RADS criteria 8. MRI images were acquired on different scanners at the 35 referring region medical centers. All examination protocols included a T2-weighted and/or STIR sequence as well as T1-weighted contrast-enhanced dynamic images before and after single-dose Gd-based contrast media injection at 1.5 or 3 T, all in line with EUSOBI and EUSOMA recommendations 13,14. Based on the MRI, the radiologists classified the degree of suspicion of breast lesions according to the BI-RADS criteria 8. All radiologists completed real-name registration on the website (www.nuqcc.cn), and all the patients' images were uploaded. All the data and images from the website were separately reviewed by three experienced radiologists in our hospitals. In cases involving a discrepancy a consensus was reached after discussion.

### Statistical analysis

Quantitative data are presented as the means ± standard deviations (SDs). Qualitative data are presented as frequencies. To assess the correlations between features and axillary lymph node metastasis, the χ2-test was used. The sensitivity, specificity, positive predictive value (PPV), negative predictive value (NPV) and accuracy were calculated through a comparison with the pathological findings. Receiver operating characteristic (ROC) curve analysis was performed. A P value <0.05 was considered statistically significant. Statistical analyses were performed with SPSS software (Version 19.0, SPSS Chicago, IL, USA) and MedCalc 11.4.2.0 software (MedCalc Software, Ostend, Belgium).

## Results

### Clinical and pathological status of the patients

The age range of the included patients was 20 to 77 years (median, 48 years). Based on pathology, 169 cases (69.8%) had breast cancer, and 73 (30.2%) did not. Among the patients with breast cancer, 99 had invasive carcinoma, 39 had intraductal carcinoma, one had mucinous carcinoma, 2 had adenocarcinoma, 3 had medullary carcinoma, 3 had a malignant phyllodes tumor, 5 had lobular carcinoma in situ, 3 had ductal carcinoma in situ, 3 had carcinoma with neuroendocrine features, 4 had micropapillary carcinoma, 2 had tubular carcinoma, 4 had papillary carcinoma and one had lymphoma. Among the patients without breast cancer, 27 had fibrocystic disease and adenosis, 26 had fibroadenoma, 10 had intraductal papilloma, 2 had inflammatory and related lesions, one had papilloma associated with fibroadenoma, a benign phyllodes tumor, 2 had a cyst, one case of fat necrosis, one case of sclerosing adenosis, one case of benign breast tissue, and one case of stromal fibrosis [Table 1].

### Clinical and imaging characteristics of benign and malignant breast lesions

Comparisons of US and clinical features between malignant and benign lesions are shown in Table 1. There were significant differences in age (P<0.001), tumor size (P<0.001), tumor shape (P=0.012), growth orientation (P<0.001), margin (P<0.001), contour (P<0.001), microcalcifications (P<0.001), presence of architectural distortions (P<0.001), color Doppler flow (P<0.001), elastography (ES) score (P<0.001) and US BI-RADS category (P<0.001) between the malignant and benign groups. However, echogenicity (P=0.15), acoustic shadowing (P=0.51) and the presence of duct ectasia (P=0.46) were not associated with lesion malignancy [Table 1]. The overall MRI BI-RADS categories were also significantly different (P<0.001) between the malignant and benign groups.

### Diagnostic performance of US and MRI BI-RADS by category

In terms of the US BI-RADS category, the ROC curves demonstrated that the best cut-off value was US BI-RADS 4b. The sensitivity, specificity, PPV, NPV, accuracy and AUC were 94.7%, 90.7%, 95.8%, 88.0%, 93.4% and 0.93 (95%CI, 0.88-0.97), respectively. In terms of the MRI BI-RADS category, the ROC curves demonstrated that the best cut-off value was MRI BI-RADS 4. The sensitivity, specificity, PPV, NPV, accuracy and AUC were 98.2%, 57.5%, 84.3%, 83.3%, 86.0% and 0.78 (95%CI, 0.71-0.85), respectively. When combining the optimal US BI-RADS and MRI BI-RADS categories, the sensitivity, specificity, PPV, NPV, accuracy, and AUC were 92.9%, 91.8%, 96.3%, 84.8%, 92.6% and 0.93 (95%CI, 0.88-0.97), respectively.

The diagnostic value of the US BI-RADS category was better than that of the MRI BI-RADS category alone (AUC 0.93 vs 0.78, P<0.001). The US BI-RADS category showed a significantly higher specificity than the MRI BI-RADS category (90.7% vs 57.5%, P<0.001), whereas the MRI BI-RADS category yielded a higher sensitivity (98.2% vs 94.7%, P=0.043). The diagnostic value of combining the US BI-RADS with the MRI BI-RADS category is equal to the US BI-RADS stratification alone (AUC 0.93 vs 0.93, P=0.81) [Table 2].

## Discussion

Dense breasts can hide nodules on mammography, and a negative result on mammography does not reliably exclude the presence of breast lesions. The most widely available supplemental screening options for patients with dense breast tissue are US and MRI, but there has been a lack of information to guide the decision to utilize one or the other versus both. The results of the present study showed that US showed a higher specificity for detecting malignant breast lesions, whereas MRI yielded a higher sensitivity. Overall, US showed better diagnostic efficiency in differentiating nodules in dense breasts than MRI. The results also showed that the diagnostic value of US and MRI combined was equal to the value of US alone.

US has been applied to more than 200,000 women and is capable of increased lesion detection in addition to mammography for women with dense breasts, similar to the results of this study 15-16. Among the conventional US characteristics of breast lesions, this study showed that irregular tumor shape, nonparallel growth orientation, indistinct margin, angular contour, microcalcification and color Doppler flow on US imaging were significantly related to breast cancer in dense breasts. However, there are several barriers to implementing screening US in practice. One of these has been the high rates of false positives from US. A recent study showed a PPV of 48% for biopsies performed based on the results of screening US in women at average risk for breast cancer with dense breasts 17.

The revisions to the BI-RADS Fifth Edition (2013) grant ES a complementary role in the ultrasonic diagnosis of breast nodules. A 5-point scale was adopted according to the hardness of the nodules in strain elastography 18. Previous studies have reported an increase in accuracy when combining B-mode US and ES 19-20. A previous study reported that the addition of elastography and color Doppler US to B-mode US in dense breasts increased the AUC to 0.96 and specificity to 76.4% without loss of sensitivity 10. In this study, similarly, the highest AUC and specificity of US were achieved when elastography was added to conventional US for diagnosing breast cancer in dense breasts. Moreover, the fifth BI-RADS category had a PPV of 95.8%, and the high PPV helped reduce the number of false-positive findings without missing cancers. A relatively high PPV helps reduce unnecessary biopsies.

For high-risk women of any breast density, supplemental screening with annual MRI has been proven to reduce late-stage disease and increase metastasis-free survival 21-22. According to a systematic review, the use of MRI for high-risk women improved the sensitivity of lesion detection by mammography from 32 to 84% 23. Moreover, the use of supplemental MRI screening in women with extremely dense breast tissue and normal results on mammography resulted in the diagnosis of significantly fewer interval cancers than mammography alone 24-25. MRI has been considered the most sensitive screening modality for women with dense breasts 26. In this study, MRI also performed well in differentiating breast nodules in dense breasts, exhibiting a higher sensitivity (98.2%) than US. Previous studies have shown that conventional ultrasound did not improve detection over MRIs and that conventional US leads to a greater number of false-positive breast cancers compared to MRI 27. In this study, combined with ES, US showed higher specificity and had a better diagnostic efficiency in differentiating nodules in dense breasts than MRI.

There are several limitations to the study. First, the 35 hospitals are referral cancer centers instead of community hospitals, which may have resulted in an increase in the malignancy ratio of the nodules in the patient population. Second, all of the patients had pathology results. Therefore, the study included more malignant breast nodules than benign nodules, which may have led to selection bias and resulted in the underestimation of the NPV and the overestimation of the PPV. Third, potential biases may have been present because the many lesions detected during the study period were not subjected to MRI examinations, as these were performed at the discretion of the patient. Fourth, because 35 radiologists performed the MRI and US examinations, there may have been interobserver differences.

In conclusion, this study found that tumor shape, growth orientation, margin, microcalcification, color Doppler flow and ES score on US were independently associated with breast cancer in patients with dense breasts. US BI-RADS showed a higher specificity, and MRI yielded a higher sensitivity. US BI-RADS had a better diagnostic efficiency in differentiating nodules in dense breasts than MRI. For the diagnosis of malignancy in patients with dense breasts, MRI and US BI-RADS are supplemental diagnostic tools to detect lesions, with US BI-RADS being the preferred adjunctive resource.

## Figures and Tables

**Figure 2:**
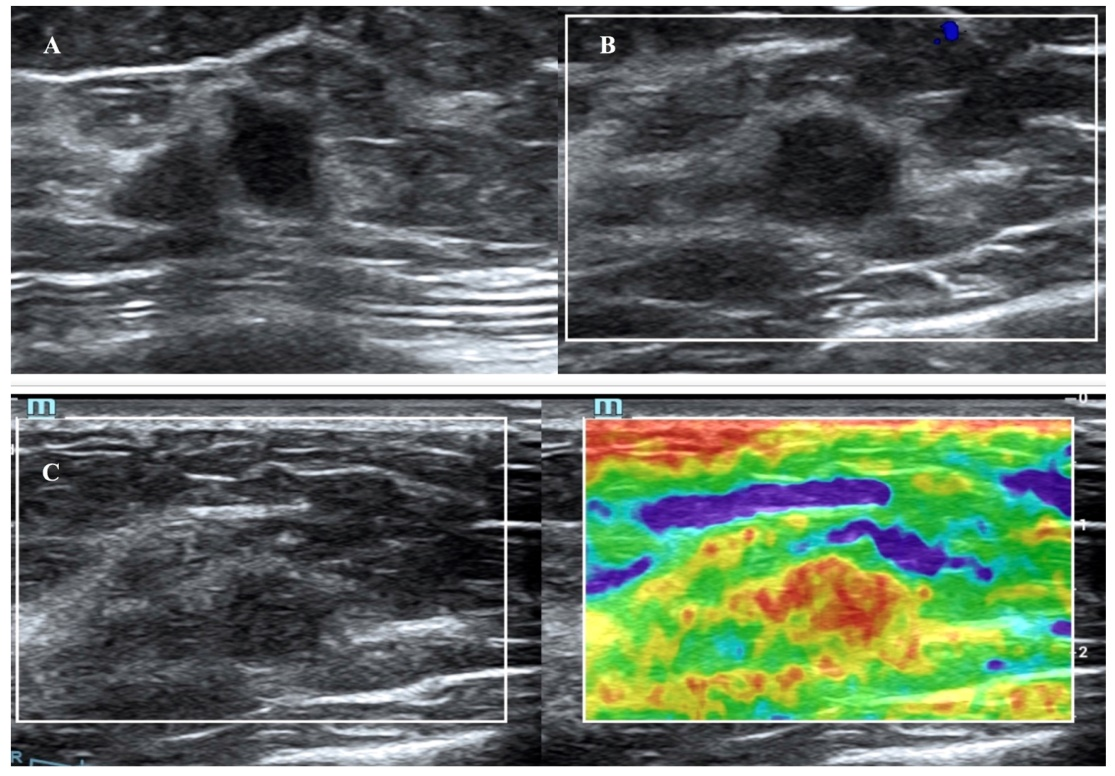
A breast mass from a 64-year-old woman with invasive carcinoma, which was classified as US BI-RADS category 4b and MRI BI-RADS category 3. B-mode US showed a 1.3-cm hypoechoic mass (A). The color Doppler ultrasound image of the same mass reveals no internal or peripheral blood flow signal (B). The elastographic image showed that the lesion was scored as a 5 with the 5-point method (C).

**Table 1:** Clinical and imaging characteristics of benign and malignant breast lesions

	Benign, n (%)	Malignant, n (%)	Total	P
Age	44.5±9.4	49.3±10.6		0.001
				
Age				<0.001
<50	55(39.0)	86(61.0)	141	
≥50	18(17.8)	83(82.2)	101	
First-degree relatives with breast cancer				0.70
0	73(30.3)	168(69.7)	241	
1	0(0.0)	1(100.0)	1	
Tumor size (cm)				<0.001
<2	68(57.1)	51(42.9)	119	
≥2	15(12.2)	108(87.8)	123	
Tumor shape				0.012
Round, oval	73(31.7)	157(68.3)	230	
Irregular	0(0.0)	12(100.0)	12	
Growth orientation				<0.001
Parallel	68(37.0)	116(63.0)	184	
Nonparallel	5(8.6)	53(91.4)	58	
Margin				<0.001
Circumscribed	22(12.0)	162(88.0)	184	
Indistinct	51(87.9)	7(12.1)	58	
Contour				<0.001
Smooth, lobulated	66(45.8)	78(54.2)	144	
Angular	7(7.1)	91(92.9)	98	
Acoustic shadowing				0.51
Yes	11(28.9)	27(71.1)	38	
NO	62(30.4)	142(69.6)	204	
Microcalcification				<0.001
Yes	11(28.9)	27(71.1)	38	
NO	62(30.4)	142(69.6)	204	
Echogenicity				0.15
Hypo-echoic	63(28.9)	155(71.1)	218	
Complex	10(41.7)	14(58.3)	24	
Presence of architectural distortion				<0.001
Yes	0(0.0)	33(100.0)	33	
No	73(34.9)	136(65.1)	209	
Presence of duct ectasia				0.46
Yes	5(26.3)	14(73.7)	19	
No	68(30.5)	155(69.5)	223	
CDFI level				<0.001
0-1	54(57.4)	40(42.6)	94	
2-3	19(12.8)	129(87.2)	148	
ES score				<0.001
1	5(71.4)	2(28.6)	7	
2	22(75.9)	7(24.1)	29	
3	36(48.6)	38(51.4)	74	
4	10(11.2)	79(88.8)	89	
5	0(0.0)	43(100.0)	43	
ES score				<0.001
1-3	63(57.3)	47(42.7)	110	
4-5	10(7.6)	122(92.4)	132	
US BI-RADS category				<0.001
2	1(100.0)	0(0.0)	1	
3	26(96.3)	1(3.7)	27	
4a	39(83.0)	8(17.0)	47	
4b	5(16.1)	26(83.9)	31	
4c	2(2.6)	76(97.4)	78	
5	0(0.0)	58(100.0)	58	
US BI-RADS category				<0.001
3-4b	66(88.0)	9(12.0)	75	
4b-5	7(4.2)	160(95.8)	167	
MRI BI-RADS category				<0.001
1	4(80.0)	1(20.0)	5	
2	8(100.0)	0(0.0)	8	
3	30(93.8)	2(6.3)	32	
4	31(20.8)	118(79.2)	149	
5	0(0.0)	48(100.0)	48	
MRI BI-RADS category				<0.001
0-3	42(93.3)	3(6.7)	45	
4-5	31(15.7)	166(84.3)	197	

Abbreviations: BI-RADS: Breast Imaging-Reporting and Data System; US: ultrasound; MRI: magnetic resonance imaging; ES: elastography; CDFI: color doppler flow imaging

**Table 2:** Diagnostic efficiency of US and MRI BI-RADS for predicting breast cancer in dense breasts

	Sensitivity (%)	Specificity (%)	PPV (%)	NPV (%)	Accuracy (%)	AUC (95%CI)
MRI BI-RADS category	98.2	57.5	84.3	83.3	86.0	0.78 (0.71-0.85)
US BI-RADS category	94.7	90.7	95.8	88.0	93.4	0.93 (0.88-0.97)
MRI and US BI-RADS category	92.9	91.8	96.3	84.8	92.6	0.93 (0.88-0.97)

Abbreviations: BI-RADS: Breast Imaging-Reporting and Data System; US: ultrasound; MRI: magnetic resonance imaging

## Abbreviations

BI-RADS: Breast Imaging-Reporting and Data System
ACR: American College of Radiology
US: ultrasound
MRI: magnetic resonance imaging
PPV: positive predictive value
NPV: negative predictive value
ROC: receiver operating curve
ES: elastography
SD: standard deviation
CDFI: color doppler flow imaging
